# Characterization of *Pseudomonas* sp. NIBR-H-19, an Antimicrobial Secondary Metabolite Producer Isolated from the Gut of Korean Native Sea Roach, *Ligia exotica*

**DOI:** 10.4014/jmb.2208.08043

**Published:** 2022-10-21

**Authors:** Sungmin Hwang, Jun Hyeok Yang, Ho Seok Sim, Sung Ho Choi, Byounghee Lee, Woo Young Bang, Ki Hwan Moon

**Affiliations:** 1 Clean Energy Research Center, Korea Institute of Science and Technology (KIST), Seoul 02792, Republic of Korea; 2 Laboratory of Marine Microbiology, Division of Convergence on Marine Science, Korea Maritime and Ocean University, Busan 49112, Republic of Korea; 3 Department of Marine Bioscience and Environment, Korea Maritime & Ocean University, Busan 49112, Republic of Korea; 4 National Institute of Biological Resources, Environmental Research Complex, Incheon 22689, Republic of Korea

**Keywords:** Antibiotics, *Ligia exotica*, secondary metabolite, symbiotic bacterium, *Pseudomonas* sp.

## Abstract

The need to discover new types of antimicrobial agents has grown since the emergence of antibiotic-resistant pathogens that threaten human health. The world’s oceans, comprising complex niches of biodiversity, are a promising environment from which to extract new antibiotics-like compounds. In this study, we newly isolated *Pseudomonas* sp. NIBR-H-19 from the gut of the sea roach *Ligia exotica* and present both phenotypes and genomic information consisting of 6,184,379 bp in a single chromosome possessing a total of 5,644 protein-coding genes. Genomic analysis of the isolated species revealed that numerous genes involved in antimicrobial secondary metabolites are predicted throughout the whole genome. Moreover, our analysis showed that among twenty-five pathogenic bacteria, the growth of three pathogens, including *Staphylococcus aureus*, *Streptococcus hominis* and *Rhodococcus equi*, was significantly inhibited by the culture of *Pseudomonas* sp. NIBR-H-19. The characterization of marine microorganisms with biochemical assays and genomics tools will help uncover the biosynthesis and action mechanism of antimicrobial metabolites for development as antagonistic probiotics against fish pathogens in an aquatic culture system.

## Introduction

Antibiotics have played an important role in bacterial infectious disease control in public health [1, 2]. However, antibiotic-resistant bacteria have been a significant emerging global threat over the past decade [1, 3]. Since 2017, the World Health Organization has published a “priority pathogens” list, emphasizing the threat of bacteria to prevent further emergence of antibiotic-resistant bacteria and encourage the development and research of new classes of antibiotics [4, 5]. Despite recognizing the serious problems of drug resistance, the major factors responsible for the rise of drug-resistant bacteria are severe misuse and overuse of antibiotics by humans [2]. Furthermore, the research and development of new classes of antibiotics against pathogens have been delayed due to economic and regulatory obstacles during the past decade [1, 3]. A number of healthcare approaches are applied in many countries to reduce and limit the spread of antibiotic resistance. In addition, various alternative strategies and control methodologies have been proposed for control of antibiotic-resistant bacteria with new concepts (*e.g.*, bacteriophage therapy, microbiome therapy, immunotherapy) [6-8], new targets (*e.g.*, teixobactin) [9], new methods (*e.g.*, AI and machine learning approach) [10], and new sources.

The ocean is a promising platform for discovering new antimicrobial agents. Classic research for the discovery of antimicrobial compounds has been conducted directly in marine animals, marine plants, and marine environmental sources [11, 12]. However, the exploration of natural chemical compounds from host microbiomes, such as gut microflora in animals or symbiotic bacteria in plants, is considered a new paradigm for antimicrobial drug discovery [3]. Many symbiotic bacteria produce beneficial secondary metabolite compounds (*e.g.*, bacteriocin, fatty acid, polyamine) that can be biodegradable, antimicrobial, and have immune-boosting activities [13].

Aquatic decomposers would be one of the potential organisms to harbor symbiotic bacteria since such scavengers play an important ecological role by recycling organic matter in decomposing waste, plants, and animal carcasses and releasing nutrients into the ecosystem [14]. The sea roach, *Ligia exotica*, is a marine-borne decomposing organism that inhabits supralittoral and intertidal zones worldwide, where during its entire lifecycle it encounters pathogens brought by pollution from both marine and terrestrial sources [15]. Unlike vertebrates, invertebrates, including the sea roach, employ various defense approaches against pathogens, such as mRNA and protein modification and genomic diversification [16].

The increase of emerging antibiotic-resistant bacteria and microbial infection demands the discovery and development of alternative treatments. To this end, we found a novel strain, *Pseudomonas* sp. NIBR-H-19, isolated from the gut of *L. exotica*, which was captured off the coast of Yeoungdo-gu, Busan, Korea (35°04'40.0"N, 129°05'20.1"E) in April, 2019, and shows antimicrobial activity against *Staphylococcus aureus*, *Streptococcus hominis* and *Rhodococcus equi*, which are representative pathogenic bacteria. Additionally, our in silico analysis identified nine secondary metabolite biosynthesis gene clusters harboring potential compounds with antimicrobial activity. Overall, this study proposes uniquely that whole genome sequencing data derived from symbiotic bacteria in marine-borne organisms provide us with valuable information regarding new candidate antimicrobial agents.

## Materials and Methods

### Host Collection

Sea roaches were collected from coastal waters near Dongsam-dong, Yeongdo-gu, Busan, Korea (GPS coordinates 35°04'39.8"N 129°05'19.1"E) in April, 2019, and deposited in a pre-sterilized container until arrival at the laboratory.

### Bacteria Isolation

For bacterial isolation, a sea roach was transferred into a 50 ml conical tube and filled with 10 ml of 70% ethanol, followed by mixing gently by inversion for 1 min. Then, 10 ml of sterile phosphate-buffered saline (PBS) was added to the ethanol-washed sample for rinsing twice. After external sterilization, 5 ml of PBS was added to the tube and the washed sea roach was ground-up by a homogenizer. The slurry (100 μl) was transferred and spread onto Brain Heart Infusion (BHI) agar (Difco Inc., USA), followed by incubation at 27°C for 24 h. Individual colonies of various morphology were selected and purified. Isolated bacteria were store at -70°C with 20% glycerol stock solution until needed. Bacterial strains with antimicrobial activity (see below Antibacterial activity characteristics) against 25 types of pathogen were chosen for further study.

### Antibacterial Activity Characteristics

A modified disc diffusion assay was used to screen the isolates, which have antibacterial activity against 25 types of pathogen (Fig. S1). Briefly, liquid culture of the pathogens was prepared after the incubation at optimal temperature for overnight and diluted by 10^3^-fold. Then, 150 μl of liquid culture was spread with a sterilized cotton swab on solid BHI plates (90 × 15 mm, SPL Life Science, Korea). *Pseudomonas* sp. NIBR-H-19, *Pseudomonas aeruginosa*, and *Pseudomonas syringae* pv. tomato were used as experimental bacteria in the liquid cell cultures. *Escherichia coli* DH5α was used as a negative control whereas spectinomycin (5 mg/ml) or gentamicin (5 mg/ml) were used as a positive control. The liquid cell cultures or antibiotics (10 μl) were dropped onto plates on which pathogens were spread and dehydrated, followed by incubation at 27°C for 72 h.

### Strain Characterizations

API 20E, API 20 NE, and API ZYM systems (BioMerieux, France) were used to test the biochemical characteristics according to the manufacturer's instructions. In addition, in biological triplicates, the bacterial growth rate at different sodium chloride concentrations was determined using 200 μl of Luria-Bertani (LB) broth by adding 0 to 5% NaCl (at 1% intervals, w/v). pH tolerance range was investigated using 200 μl of LB broth with different pH values from 2 to 10 (with 1 unit pH intervals), prepared using 1 M HCl and 1 N NaOH. Then, 1% pre-cultured broth was inoculated into the LB broth and incubated at 27°C. The optical density at 600 nm was measured using an Epoch2 microplate reader (BioTek, USA) after 48 h incubation. Rolling regression was used to calculate the specific growth rate at exponential phase [17].

### Genome Sequencing and Analysis

Genomic DNA was extracted using a HiGene Genomic DNA Prep Kit (Biofact, Korea) and utilized for the library construction of whole genome sequencing using a SMRTbell Template Prep Kit 1.0 (Pacific Biosciences, USA). The raw sequencing reads obtained through a PacBio RS II single-molecule real-time cell platform (Pacific Biosciences) were assembled by the hierarchical genome assembly process (HGAP, Version 2.3) workflow including multiple rounds of polishing with Quiver [18]. Putative gene coding sequences (CDSs) from the assembled contig were identified using Glimmer v3.02 [19], and open reading frames (ORFs) were obtained. Furthermore, the ORFs were searched against the NCBI Non-Redundant protein database for all species using the Blastall alignment tool (https://www.ncbi.nlm.nih.gov/books/NBK279690/), and the best BLAST result hits were submitted to identify GO annotation by Blast2GO software [20]. In addition, rRNAs and tRNAs were predicted using RNAmmer 1.2 and tRNAscan-SE 1.4, respectively [21, 22]. antiSMASH v5.1.2 was adopted to predict genes or clusters involved in the secondary metabolism [23]. To calculate the genome similarity, the average nucleotide identity (ANI) value was measured in a web tool (http://enve-omics.ce.gatech.edu/ani/).

### Phylogenetic Analysis

The 16S ribosomal RNA gene sequence was retrieved from the complete genome of *Pseudomonas* sp. NIBR-H-19 and KEGG database (https://www.genome.jp/kegg/genome/) for the representative species in Pseudomonadales. Multiple sequences aligned by the MUSCLE (http://www.drive5.com/muscle/) were submitted to construct phylogenetic trees based on the maximum-likelihood and neighbor-joining methods coupled with the Kimura two-parameter model or the maximum parsimony with the inference of Subtree-Pruning-Regrafting method under the MEGA 11 platform [24]. Alternatively, to generate a phylogenetic tree, the genome of *Pseudomonas* sp. NIBR-H-19 was added to the Insert Genome Into SpeciesTreev2.2.0 in KBase [25]. For the estimation of confidence for the tree topology, 1,000 bootstrap replications were applied.

### Statistical Analysis

The assays and analyses were conducted in biological triplicates and the results were presented as mean values ± standard error of the mean (SEM). Significance of each variable was determined using the unpaired two-sided Student's *t*-test in R (v4.1.0.).

### Genome Sequence and Strain Accession of *Pseudomonas* sp. NIBR-H-19

The whole genome sequence of *Pseudomonas* sp. NIBR-H-19 was deposited in GenBank under the accession number CP089304 (https://www.ncbi.nlm.nih.gov/nuccore/CP089304). This strain is available from the National Institute of Biological Resources in Korea under accession number AZIQBAC000000019.

## Results

### Isolation and Physiological Characteristics

**Fig. 1:**
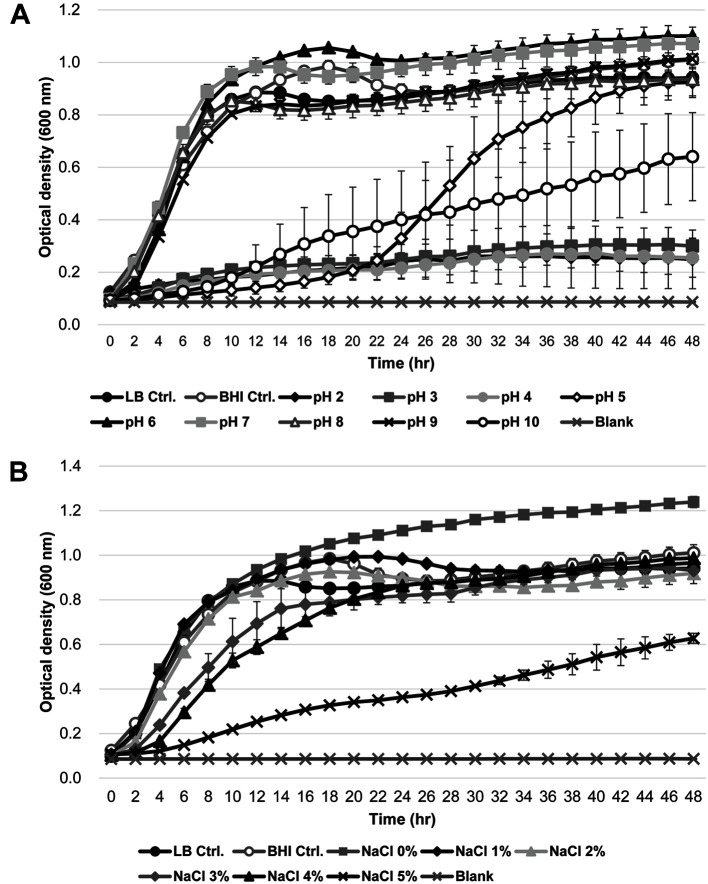
The growth of NIBR-H-19 strain at different pH and salinity. (**A**) The effect of various pH ranges on growth of NIBR-H-19 in LB broth after 48 h incubation at 27°C. (**B**) The effect of different NaCl concentrations on growth of NIBR-H-19 in LB broth with different NaCl concentrations after 48 h incubation at 27°C. (**C**) Comparison of the specific growth rates on various pH ranges. (**D**) Comparison of the specific growth rates on different NaCl concentrations. A total of *n* = 3 samples was tested. Error bars represent the standard deviation of the mean. *p < 0.05, **p < 0.01, ***p < 0.001, ****p < 0.0001.

Hundreds of isolates were isolated from the gut of the sea roach *L. exotica*. A few of the isolates were inoculated in BHI medium and dropped into culture of representative gram-negative or gram-positive strains of *E. coli* and *S. aureus*, respectively, to determine the influence on cell growth. Through this screening, we found that NIBR-H-19 inhibited the growth of *S. aureus* (Fig. S1). Hence, the strain NIBR-H-19 was selected for further biochemical characterization. NIBR-H-19 was found to be aerobic, gram-negative, motile, and rod-shaped (Table S1). The colony shape was round when grown on BHI agar at 27°C for 24 h. The NIBR-H-19 strain was able to grow on several types of media, including BHI, LB, Tryptic Soy Agar (TSA), Starch-Yeast extract-Peptone (SYP) agar, and Marine Agar (MA); however, the strain could not grow on De Man, Rogosa and Sharpe (MRS) agar medium. The ability of the NIBR-H-19 strain to tolerate different pH ranges and NaCl concentrations was investigated in LB broth for 48 h (Figs. 1 and 1B). The specific growth rate of NIBR-H-19 was improved as pH increased and the optimal range was pH 6 and 9 (Fig. 1C). In addition, the NIBR-H-19 strain showed abilities to grow at 0 to 5% of NaCl concentration (Fig. 1B). However, the specific growth rate started to significantly decrease from 3% of NaCl (Fig. 1D) [26-30]. In addition, the NIBR-H-19 strain was positive for β-glucosidase (esculin hydrolysis), protease (gelatin hydrolysis) and the assimilations for D-glucose, L-arabinose, D-mannitol, potassium gluconate, capric acid, adipic acid, malic acid and trisodium citrate, whereas it was negative for nitrates reduction, indole production, glucose fermentation, arginine dihydrolase, urease, β-galactosidase, and the assimilations for D-mannose, N-acetyl-glucosamine, D-maltose, adipic acid and phenylacetic acid (Table 1, Table S1). According to API ZYM test, strain NIBR-H-19 showed positive results for leucine arylamidase, acid phosphatase, and naphtol-AS-BI-phosphohydrolase. A weakly positive result was observed for the production of esterase (C4) and esterase lipase (C8).

### Whole Genome Sequencing and Analysis

**Fig. 2:**
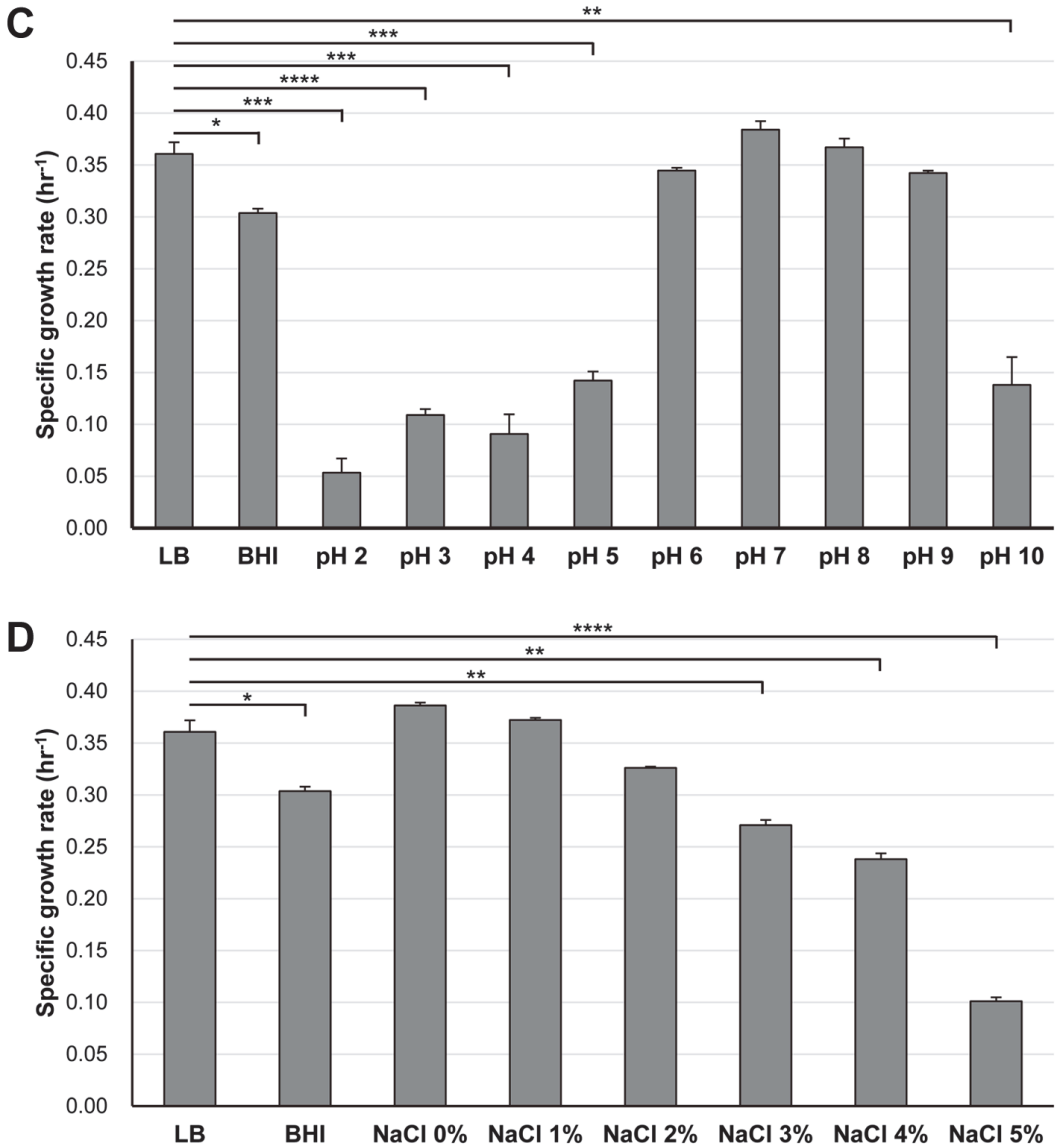
The genomic features of *Pseudomonas* sp. NIBR-H-19. Labeling from the outside to the core is as follows: forward open reading frames (ORF) (blue), reverse ORF (red), CDS (purple), rRNA (orange), tRNA (green), GC content values and GC skew (red and blue).

**Fig. 3:**
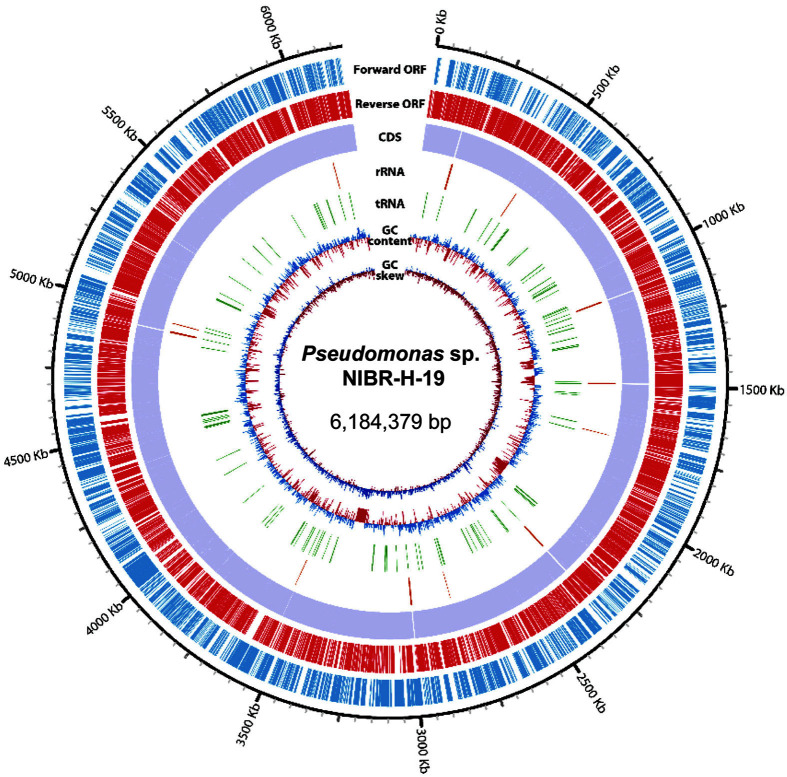
Gene ontology classification of *Pseudomonas* sp. NIBR-H-19. The number of transcripts with GO terms is provided for each of the three GO categories (biological process, cellular component and molecular function).

Next, the strain NIBR-H-19 was further characterized by applying whole genome sequencing. A total of 151,602 long reads containing 1,319,585,532 bp were obtained and further de novo assembled, resulting in one contig with an N50 value of 6,184,379 bp and an average coverage of 133X. The complete genome of NIBR-H-19 comprised a circular chromosome of 6,184,379 bp with 58.65% G+C content; however, a plasmid genome was not predicted (Fig. 2). Besides the prediction of 19 rRNAs and 75 tRNAs from the draft genome, a total of 5,644 CDSs (Table S3) were predicted and further categorized via GO analysis (Fig. 3).

**Fig. 4:**
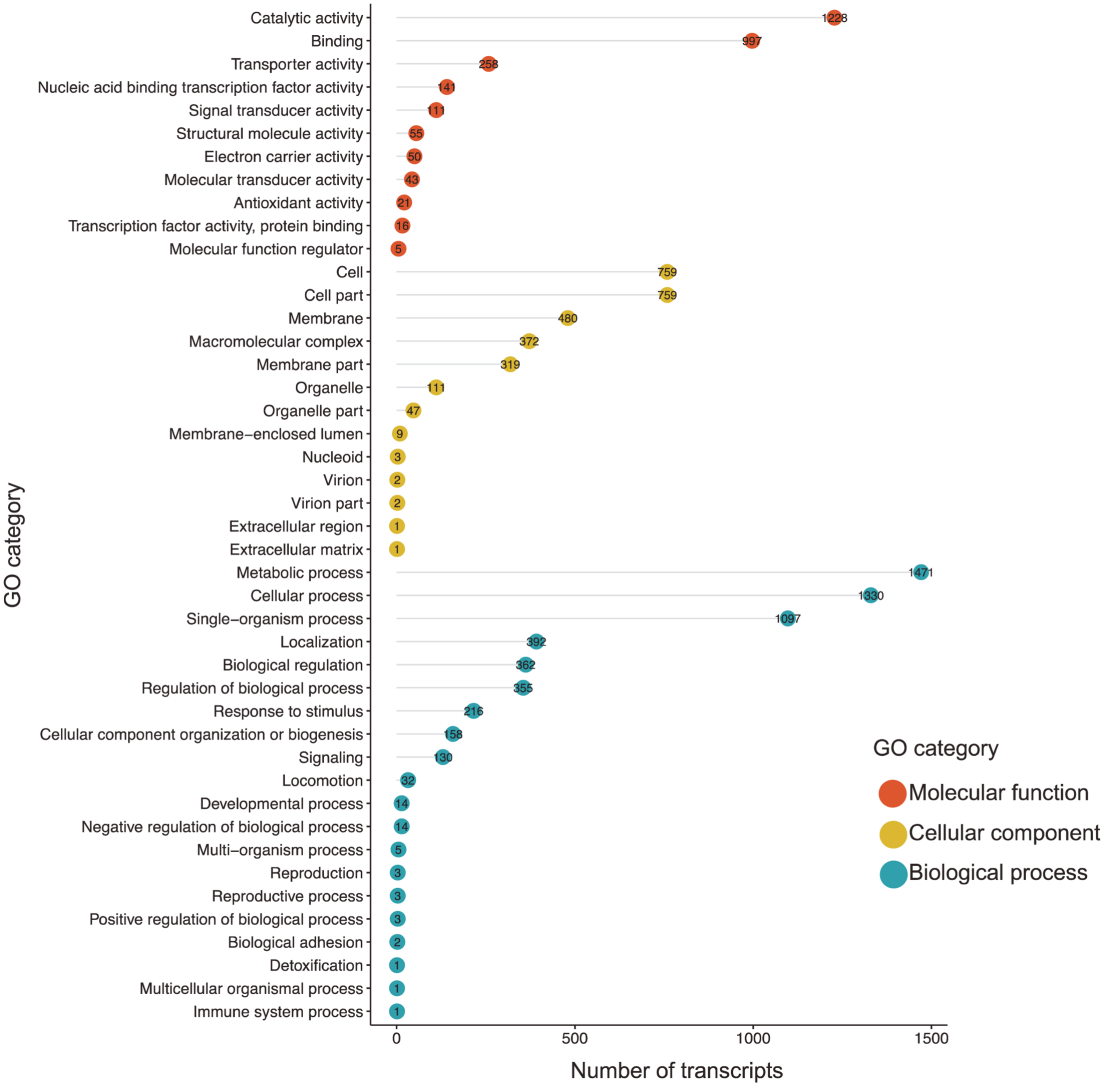
Phylogenetic trees of *Pseudomonas* sp. NIBR-H-19. (**A**) An evolutionary relationship of *Pseudomonas* sp. NIBR-H-19 to the closely related genomes. A set of 49 core, universal genes defined by COG (Clusters of Orthologous Groups) gene families were considered to construct a phylogenetic tree by the Insert Genomes into Species Tree App (The Department of Energy Systems Biology Knowledgebase, KBase). Bootstrap values are written in the percentage of 1,000 replicates and the values above 90% are denoted. (**B**) A phylogenetic tree with 16s ribosomal RNA sequences by the maximum-likelihood method. Representative species involved in the order of Pseudomonadales are indicated. Filled and empty boxes show the families of *Pseudomonadaceae* and *Marinobacteraceae*, respectively. Bootstrap values are written in the percentage of 1,000 replicates and the values above 65% are shown.

To investigate the evolutionary relatedness with other taxa, the genome of NIBR-H-19 was compared based on a set of 49 core universal genes that are parameterized in the SpeciesTree (v2.2.0) in the KBase environment [25], and the resulting phylogenetic tree showed that the NIBR-H-19 strain is a member of *Pseudomonas* species (Fig. 4A). Next, we extracted the sequence of 16S ribosomal RNA from the NIBR-H-19 strain and further compared it with the representative species in Pseudomonadales. Among the two families *Pseudomonadaceae* and *Marinobacteraceae*, the newly isolated microorganism was clustered in *Pseudomonadaceae* and the closest species was *Pseudomonas fluorescens* Pf0-1, suggesting that the NIBR-H-19 strain is a bona fide *Pseudomonas* member (Figs. 4B and S2). In addition, the average nucleotide identity (ANI) value between *Pseudomonas* sp. NIBR-H-19 and the closest strains of *P. koreensis* and *P. fluorescens* Pf0-1 were 86.6 and 84.4%, respectively. These ANI values are lower than the cut-off (95–96%) to evaluate the delineation of different bacterial species. Taken together, these results support that the newly isolated NIBR-H-19 is an unexplored *Pseudomonas* species.

### Putative Biosynthetic Gene Clusters for Secondary Metabolites

Since the gut is one of the most complex niches comprising myriad microbial consortia, we sought the physiological characteristics of *Pseudomonas* sp. NIBR-H-19, especially focusing on the production of secondary metabolites, which could act as potential antibiotics. To this end, the whole genome of *Pseudomonas* sp. NIBR-H-19 was scanned using antiSMASH v5.1.2 [23]. A total of nine regions were predicted to synthesize six different secondary metabolite types (Table 2). Of these, two regions (#3 and #9) that may synthesize antimicrobial peptides could be involved in antimicrobial activity. Lanthipeptide is a representative small ribosomally synthesized and post-translationally modified peptide that shows excellent antibiotic activity against gram-positive microorganisms, such as methicillin-resistant *S. aureus*, *Streptococcus pneumoniae*, vancomycin intermediate *S. aureus*, and *Clostridioides difficile* [31]. The lanthipeptide antibiotic mechanism of action generally involves obstruction of cell membrane construction through pore formation or inhibition of peptidoglycan biosynthesis. In addition, *Pseudomonas* sp. NIBR-H-19 possesses a beta-lactone, fengycin-type lipopeptide (Table 2, #9). Although fengycin has been mostly reported as a potent antifungal lipopeptide, its antibacterial activity was also recognized [32]. Fengycin functions similar to lanthipeptide by disturbing the cell membrane to inhibit bacterial growth; however, it is formed non-ribosomally via a large multifunctional enzyme complex [33]. Furthermore, it is effectual on blocking quorum-sensing of gram-positive bacteria [34]. Besides the two antimicrobial peptides, enzymes which may be able to synthesize other bioactive peptides, such as arylpolyene, pyoverdin, and methanobactin, were also predicted in the isolated genome. However, these small peptides are considered to be involved in stress responses, such as metal (iron or copper) homeostasis and protection from oxidative stress [35, 36].

### Characterization of Antibacterial Activity

**Fig. 5:**
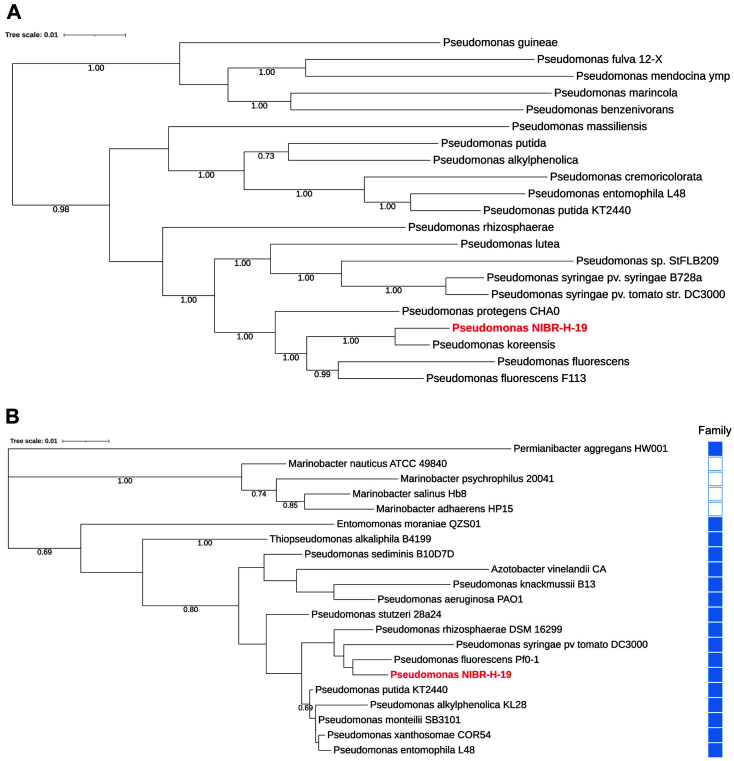
A modified disc diffusion assay for the antibacterial activity. (**A**) Schematic of the assay. 200 μl of prey cells culture were spread on the whole plate and 20 μl of cell culture of *Pseudomonas* sp. NIBR-H-19, *P. aeruginosa*, *P. syringae* pv. tomato, *E. coli* DH5α, BHI medium, and spectinomycin (final 5 μg/ml) were spotted as indicated. (B-D) The growth inhibition of *S. aureus* (**B**), *S. hominis* (**C**), and *R. equi* (**D**) by *Pseudomonas* sp. NIBR-H-19. Images were taken after 72 h incubation at 27°C. A white, dashed line around the *Pseudomonas* sp. NIBR-H-19 colony indicates the growth inhibition clear zone area.

During the bacterial isolation, *Pseudomonas* sp. NIBR-H-19 displayed antimicrobial activity against *S. aureus*. To examine further antimicrobial activity of metabolites from *Pseudomonas* sp. NIBR-H-19, a drop assay was carried out with 25 different types of pathogen (Fig. S1, Table S2). The antimicrobial activities of *Pseudomonas* spp. are well-known results. Generally, three different type VI secretion systems (T6SS) are used for disabling and destruction of other bacteria in *Pseudomonas* spp. [37, 38]. In addition, some *Pseudomonas* spp. show antagonistic activity in their environment by producing siderophore and biosurfactant [39, 40]. However, the 25 different prey bacteria were not influenced by *P. aeruginosa*, *P. syringae* pv. tomato, *E. coli* DH5α, and BHI medium, and furthermore, no growth was seen in the area where antibiotic (spectinomycin or gentamicin) was spotted (Fig. 5). Interestingly, a clear halo was observed around the *Pseudomonas* sp. NIBR-H-19 culture against *S. aureus*, *S. hominis*, and *R. equi*, indicating that the growth of *Pseudomonas* sp. NIBR-H-19 causes significant growth defects to some pathogens with narrow host range. Indeed, the genomic analysis of *Pseudomonas* sp. NIBR-H-19 proved that a beta-lactone-type lipopeptide fengycin secondary metabolite would be produced, specifically targeting gram-positive bacteria [32]. This secondary metabolite was not found in the genomic analyses of *P. aeruginosa* PAO1 and *P. syringae* pv. tomato str. DC3000 (Table S4). The drop assay suggests that secondary metabolites are produced during the growth of *Pseudomonas* sp. NIBR-H-19, and that they can act as antibiotics against specific pathogens.

## Discussion

Since Sir Alexander Fleming discovered penicillin in 1928, antibiotics have been extensively utilized to cure patients of bacterial infectious diseases [41, 42]. However, the mass treatment of antibiotics without proper protocols resulted in the emergence of methicillin-resistant *S. aureus* (MRSA) that was first reported in the United Kingdom in 1961 [43, 44]. The World Health Organization (WHO) recently announced a list of drug-resistant bacteria that pose the greatest threat to human health [45]. Besides the alternative approaches, such as immunomodulation, bacteriophage therapy and probiotics, a novel and promising act would be the uncovering of new types of antimicrobial agents to control drug-resistant bacterial infections [46]. To this end, the primary objective of our study was to explore the microbial diversity inside the gut of *L. exotica* (sea roach) and to screen strains that possess antimicrobial chemicals.

We isolated hundreds of bacteria from gut extract and examined all of them for their antimicrobial activities using a modified disc diffusion assay in an agar plate. One of the isolates showed inhibitory activity against three pathogenic microorganisms including *S. aureus*, *S. hominis* and *R. equi* (Fig. 5). Interestingly, while the cell growth of gram-negative organisms, such as *Acinetobacter boumannii*, *Edwardsiella piscicida*, and *Salmonella typhi* was hardly inhibited by the cell culture of the isolate, the antimicrobial activity was observed only in gram-positive bacteria, suggesting that the isolate secretes a relatively narrow antimicrobial agent specific to gram-positives (Fig. S1). This gram-positive-specific antimicrobial agent produced from a halotolerant alkaliphilic *Streptomyces aburaviensis* Kut-8 was previously reported [47]. This may be because of the different structural composition of cell wall between gram-positives and gram-negatives. Compared to gram-negative bacteria carrying lipopolysaccharides within the outer membrane, gram-positives containing a relatively thick peptidoglycan layer would be susceptible in the presence of a gram-positive-specific antimicrobial agent.

Further analyses with biochemical assays and whole genome sequencing were performed to identify the isolate and the uniqueness compared to other strains. Based on the comparison with sequences of 16S ribosomal RNA and multivalent essential genes, the isolate from gut of sea roach is a member of *Pseudomonadaceae* and its closest species are *P. koreensis* and *P. fluorescens* Pf0-1 (Fig. 4). The isolated strain had a pairwise sequence similarity (ANI score) of 86.6 and 84.4% with *P. koreensis* and *P. fluorescens* Pf0-1, respectively, in addition to a respective score of 78.5 and 80.6% with other *Pseudomonas* strains, *P. aeruginosa* and *P. syringe*. These values show that the isolate has a distinctive evolution compared to those that are clustered in different *Pseudomonas* clades. *Pseudomonas*, currently registered in 381 genomes in the NCBI Genome database (as of October, 2022), is one among the diverse genera of prokaryotes and members of *Pseudomonas* that have been isolated from a wide variety of places, including inside living creatures, and terrestrial and aquatic surroundings. Interestingly, even many subspecies belonging to the same species encode hundreds of different genes that have not been identified from members of *Pseudomonas* [48]. Among the average encoding 5,628 genes from the four representative *Pseudomonas* species (*P. aeruginosa* PAO1, *P. fluorescens* Pf0-1, *P. putida* KT2440 and *P. syringae* pv. tomato DC3000), about 386 genes (equal to 7% of total encoding genes) are not predicted to be present in other subspecies. This suggests that new types of subspecies still exist and remain to be isolated and characterized. Overall, these genomic discrepancies account for the newly isolated strain that is distinctively evolved compared to other members of *Pseudomonas*.

Living organisms produce various types of bioactive secondary metabolites to defend themselves against environmental threats and infection by microbial pathogens [49-54]. Bacteria also produce a variety of high-value chemicals, including the unique biological functions of secondary metabolites. These bacterial secondary metabolites have aroused great pharmacological interest due to their antimicrobial activities, which have the ability to replace antibiotics in controlling drug-resistant bacteria [55]. Through the genome analysis of the isolate, among a total of nine regions were predicted to synthesize six different secondary metabolites, and two antimicrobial agents, lanthipeptide and beta-lactone (fengycin type lipopeptide), are predicted to be critical to the growth of gram-positives. These antimicrobial peptides were previously shown to exert antibiotics-like activity in *S. aureus* and *S. pneumoniae* by obstructing peptidoglycan biosynthesis during cell membrane construction or pore formation [31]. Interestingly, gene clusters for the synthesis of fengycin were not predicted in *P. aeruginosa* PAO1 and *P. syringae* pv. tomato str. DC3000, whereas they are likely present in *P. koreensis* and *P. fluorescens*, supporting the close evolution of the isolate with them (Table S4).

In this study, we newly isolated *Pseudomonas* sp. NIBR-H-19, a novel species identified from the sea roach *L. exotica*, and investigated its biochemical characteristics, as well as revealed the whole genome sequence. Our results shed light on the discovery of a new type of antibiotics by identifying the genetic and physiological basis that allows this strain to survive in the gut microbiome by producing secondary metabolites. Hence, in vivo assay will be needed in the future to validate the potential of this strain for use as an antagonistic probiotic against fish pathogens in an aquatic culture system.

## Supplemental Materials

Supplementary data for this paper are available on-line only at http://jmb.or.kr.

## Figures and Tables

**Table 1:** Physiological characteristics of the strain NIBR-H-19 and the species of the genus *Pseudomonas*.

Characteristics	1	2	3	4
NaCl tolerance (%, w/v)	1-4	1-5	N.D.	1-5
pH	5-9	N.D.	N.D.	N.D.
Temperature(°C)	4-28	4-30	4-28	4-28
Nitrates reduction (to nitrites)	-	-	N.D.	-
Nitrates reduction (to nitrogen)	-	-	N.D.	-
Indole production	-	-	-	-
Glucose fermentation	-	-	-	N.D.
Arginine dihydrolase	-	+	-	+
Urease	-	+	-	+
β-glucosidase (esculin hydrolysis)	+	-	-	-
Protease (gelatin hydrolysis)	+	+	-	+
β-galactosidase	-	-	-	-
D-glucose assimilation	+	+	+	+
L-arabinose assimilation	+	+	+	-
D-mannose assimilation	-	+	+	+
D-mannitol assimilation	+	+	+	+
N-acetyl-glucosamine assimilation	-	+	-	+
D-maltose assimilation	-	-	-	-
Potassium gluconate assimilation	+	+	+	+
Capric acid assimilation	+	+	+	-
Adipic acid assimilation	-	-	-	-
Malic acid assimilation	+	+	+	-
Trisodium citrate assimilation	+	+	+	+
Phenylacetic acid assimilation	-	-	-	-

Strains: 1, Strain NIBR-H-19; 2, *Pseudomonas koreensis* [26, 29]; 3, *Pseudomonas rhizosphaerae* [27]; 4, *Pseudomonas protegens* CHA0 [28, 30]. +, positive; -, negative; N.D., not determined.

**Table 2:** Potential production of secondary metabolites from *Pseudomonas* sp. NIBR-H-19.

Region No.	Type^a)^	From (bp)	To (bp)	Most similar compound
1	NRPS	155,374	207,366	Pyoverdin
2	NAGGN	340,361	355,243	
3	Lanthipeptide	1,919,160	1,942,093	
4	Bacteriocin	2,058,520	2,067,955	Methanobactin
5	Arylpolyene	2,641,814	2,685,418	APE Vf
6	Bacteriocin	3,734,945	3,744,124	Methanobactin
7	NRPS	4,372,452	4,437,348	Pyoverdin
8	Bacteriocin	5,877,956	5,888,795	Methanobactin
9	Betalactone	5,991,953	6,015,216	Fengycin

^a)^NRPS, Non-ribosomal peptide synthetase; NAGGN, N-acetylglutaminylglutamine amide; APE Vf, Aryl polyene from *Vibrio fischeri*.
