# Supplementary material for: Characterization of Pseudomonas sp. NIBR-H-19, an Antimicrobial Secondary Metabolite Producer Isolated from the Gut of Korean Native Sea Roach, Ligia exotica
Source: J Microbiol Biotechnol. 2022 Oct 21;32(11):1416–26. doi: 10.4014/jmb.2208.08043 (PMC9720083; doi:10.4014/jmb.2208.08043)
Supplement: Supplementary file 1 [file jmb-32-11-1416-supple.pdf]

J. Microbiol. Biotechnol.
https://doi.org/10.4014/jmb.2208.08043

Table S1. General features of *Pseudomonas* sp. NIBR-H-19 and MIGS information

**Item**	**Description**
**General features**	
Taxonomic classification	Domain Bacteria<br>Phylum Proteobacteria<br>Class Gammaproteobacteria<br>Family Pseudomonadales<br>Genus Pseudomonadaceae<br>Species *Pseudomonas* sp.<br>Strain NIBR-H-19
Gram staining	Negative
Cell shape	Rod
Motility	Motile
**MIG$_S$ data**	
Project name	Discovery for Usability of Host-defense Molecules from Biological Resources in Korea
Investigation type	Bacteria
Submitted to NCBI	PRJNA786652 (BioProject)
Geographical location	Yeongdo-gu, Busan, South Korea
Latitude and longitude	35°04'40.0"N, 129°05'20.1"E
Collection date	2019. 04. 01
Isolation source	Isolated from gut of Korea native sea roach, *Ligia exotica*
**Sequencing and genome features**	
Sequencing platform	PacBio® SMRT Sequencing
Sequencing chemistry	P6-C4 chemistry and sequenced using 1 SMRT cell with MagBead OneCellPerWell v1 Protocol
Assembler	SMRT Analysis v2.3.0 HGAP.3
Fold coverage	133 x
Genome size (bp)	6,184,379
GC content (%)	58.65
CDSs in RAST	5,644
rRNA	19
tRNA	75

Table S2. Bacteria used in this study, sensitive antibiotic, and source or reference.

Bacterial strains	Sensitive antibiotic	Source or Reference
**<u>Prey bacteria</u>**		
Acinetobacter boumannii 04 p4^{+}	Spectinomycin	Weber *et al.*[a)]
A. baumannii ATCC 17978	Gentamicin	ATCC
A. boumannii ATCC 19606	Gentamicin	ATCC
Aeromonas hydrophilla	Spectinomycin	Lab. collection
Bacillus cereus ATCC 11778	Spectinomycin	ATCC
Edwardsiella piscicida CK41	Gentamicin	Baek *et al.*[b)]
Enterococcus faecalis ATCC 10541	Spectinomycin	ATCC
Klebsiella pneumoniae ATCC 10031	Spectinomycin	ATCC
Listeria monocytogenes ATCC 15313	Spectinomycin	ATCC
Listonella anguillarum KCTC 2711	Spectinomycin	KCTC
Rhodococcus equi ATCC 6939	Spectinomycin	ATCC
Salmonella typhi ATCC 19430	Spectinomycin	ATCC
S. typhimurium χ3339	Spectinomycin	Lab. collection
Shigella flexneri ATCC 9403	Spectinomycin	ATCC
S. sonnei ATCC 29930	Spectinomycin	ATCC
Methicillin-resistant *Staphylococcus aureus* (MRSA)	Spectinomycin	Lab. collection
Staphylococcus aureus ATCC 25923	Spectinomycin	ATCC
S. haemolyticus	Gentamicin	Lab. collection
Streptococcus iniae KCTC 3657	Spectinomycin	KCTC
S. iniae GNU	Spectinomycin	Lab. collection
S. iniae NIFS	Spectinomycin	Lab. collection
Vibrio cholerae ATCC 25872	Spectinomycin	ATCC
V. parahaemolyticus ATCC 17802	Spectinomycin	ATCC
V. vulnificus ATCC 33149	Spectinomycin	ATCC
Staphylococcus hominis	Gentamicin	Lab. collection
**<u>Predator bacteria</u>**		
Pseudomonas sp. NIBR-H-19	-	This study
P. aeruginosa ATCC 27853	-	ATCC
P. syringae pv. *tomato*	-	Lab. collection
Escherichia coli DH5α	-	Promega

[a)], Proc Nat'l Acad Sci USA (2015) 112(30): 9442-7. doi: 10.1073/pnas.1502966112.

[b)], Microbiol Resour Announc (2020) 9(17): e00061-20. doi: 10.1128/MRA.00061-20.

Table S4. Potential production of secondary metabolites from *Pseudomanos* species

**Region No.**	**Type**[a)]	**From (bp)**	**To (bp)**	**Most similar compound**
Pseudomonas aeruginosa PAO1				
1	NRPS-like, betalactone	1,302,696	1,343,373	Pyoverdin
2	Hserlactone	1,549,883	1,569,709	
3	Phenazine	2,061,278	2,080,474	Endophenazine A / B
4	Thiopeptide	2,083,444	2,116,447	Oxalomycin B
5	Redox-cofactor	2,162,913	2,185,057	Lankacidin C
6	NRPS	2,513,179	2,564,072	L-2-amino-4-methoxy-trans-3-butenoic acid
7	NRPS	2,638,928	2,739,716	Pyoverdin
8	RiPP-like	3,671,227	3,681,461	
9	NRPS	3,710,749	3,757,593	
10	NAGGN	3,860,897	3,875,657	
11	Hserlactone	3,879,139	3,899,744	
12	NRPS-like	4,535,834	4,578,183	
13	RiPP-like	4,584,491	4,595,321	
14	Phenazine, NRPS	4,703,796	4,761,111	Marinophenazine A / Phenaziterpene A
Pseudomonas syringae pv. *tomato* str. DC3000				
1	Redox-cofactor	547,668	569,878	Lankacidin C
2	NAGGN	1,780,376	1,795,179	
3	NRPS	2,288,651	2,376,631	Pyoverdin
4	T1PKS, NRPS	2,854,015	2,910,581	Yersiniabactin
5	NRPS	3,133,704	3,200,018	Syringafactin
6	Hserlactone	4,367,652	4,388,386	FR901228
7	NRPS	5,068,738	5,123,916	Kanamycin
8	T1PKS, NRPS, NRPS-like	5,267,945	5,356,507	Coronatine
9	Arylpolyene	5,787,526	5,831,125	APE Vf
10	NRPS-like	6,189,553	6,233,002	Fragin
Pseudomonas fluorescens				
1	NAGGN	156,005	170,862	
2	NRPS	208,135	259,890	pyoverdin
3	siderophore	349,280	361,205	
4	redox-cofactor	1,549,767	1,571,914	lankacidin C
5	RiPP-like	2,087,249	2,096,625	
6	NRPS-like	2,288,238	2,317,494	ambactin
7	arylpolyene	2,677,089	2,720,664	APE Vf

8	RiPP-like	3,812,929	3,823,807	
9	NRPS	4,859,475	4,921,220	viscosin
10	NRPS-like	5,660,158	5,701,185	
11	thiopeptide	5,735,877	5,765,125	pseudopyronine A / pseudopyronine B
12	NRPS	5,913,902	5,979,533	pyoverdin
13	betalactone	6,289,200	6,312,353	fengycin
14	NRPS	6,415,945	6,462,214	tolaasin I / tolaasin F
Pseudomonas koreensis				
1	betalactone	307,850	331,072	fengycin
2	NRPS	504,277	564,357	pyoverdin
3	RiPP-like	1,232,470	1,241,208	
4	arylpolyene	2,131,595	2,175,199	APE Vf
5	NRPS-like	2,475,766	2,505,408	ambactin
6	RiPP-like	2,668,534	2,679,379	
7	redox-cofactor	3,209,048	3,231,204	lankacidin C
8	NAGGN	4,376,079	4,390,951	
9	NRPS	4,519,354	4,571,987	pyoverdin
10	NRPS	6,052,233	6,123,913	lokisin

[a] NRPS, Non-ribosomal peptide synthetase; NAGGN, N-acetylglutaminylglutamine amide; RiPP, Ribosomally synthesized and post-translationally modified peptide; T1PKS, Type I polyketide synthase; APE Vf, Aryl polyene from *Vibrio fischeri*

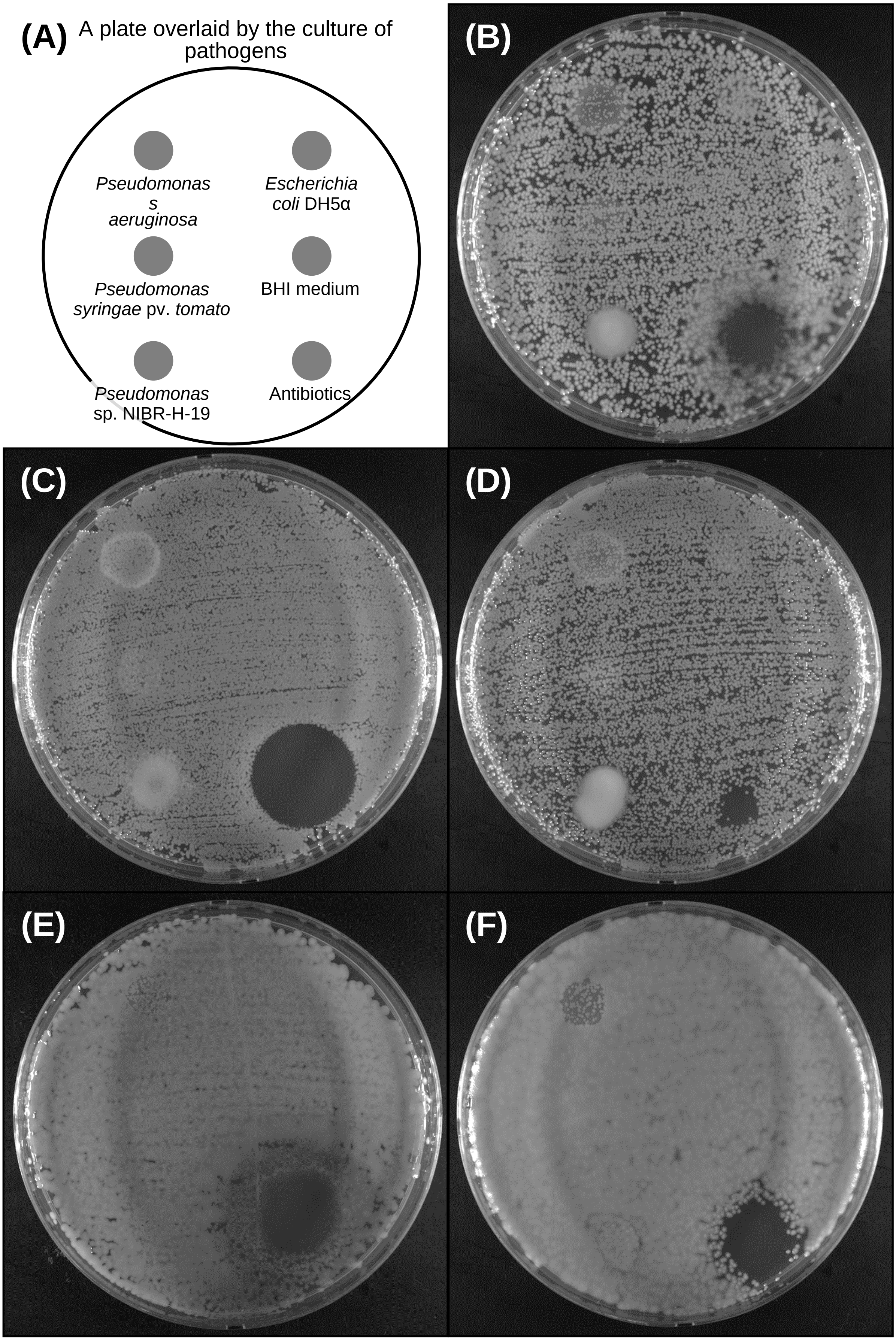
(A)
A plate overlaid by the culture of pathogens
Pseudomonas s aeruginosa
Escherichia coli DH5α
Pseudomonas syringae pv. tomato
BHI medium
Pseudomonas sp. NIBR-H-19
Antibiotics
(B)
(C)
(D)
(E)
(F)

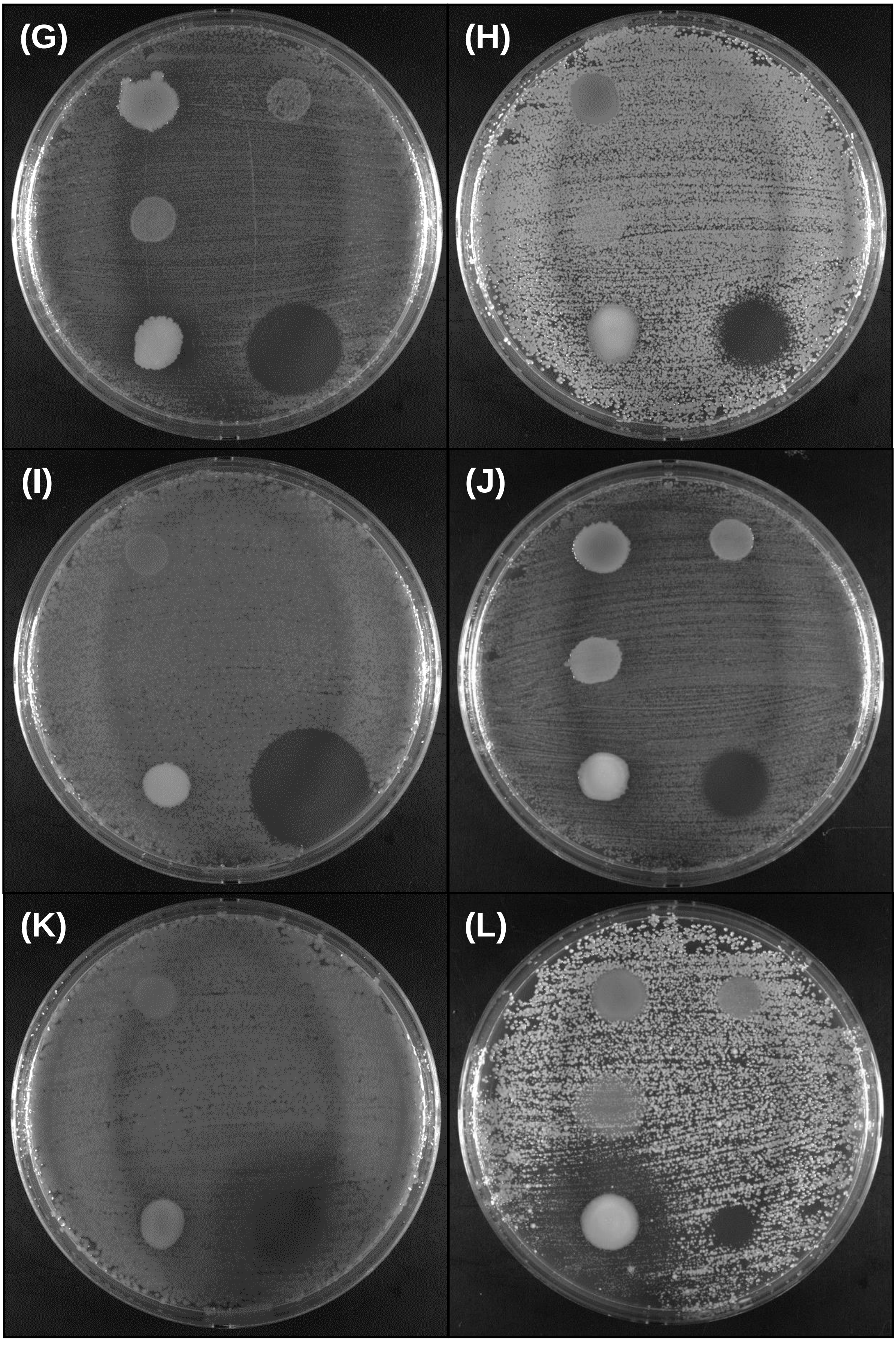
(G)
(H)
(I)
(J)
(K)
(L)

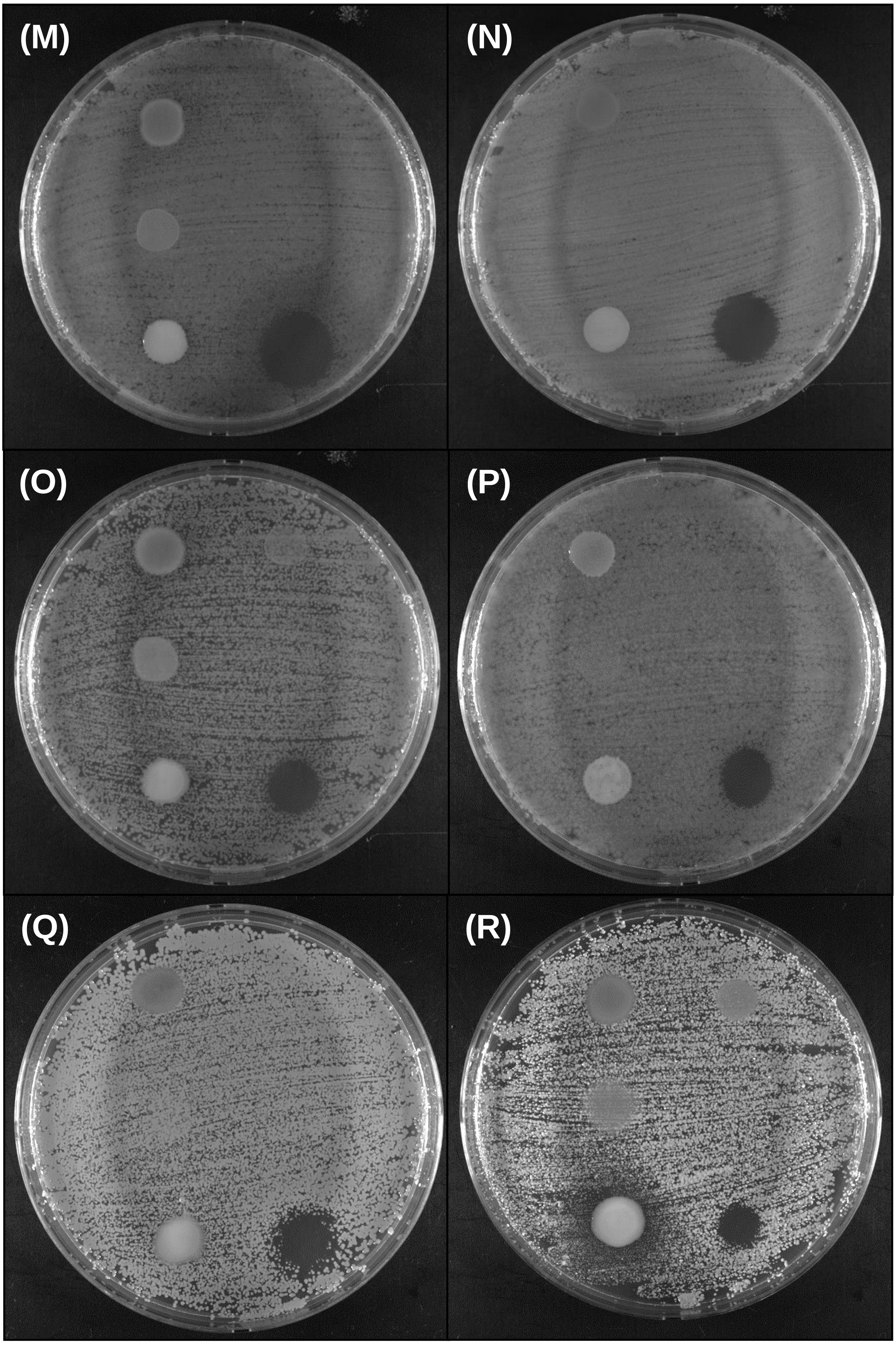
(M)
(N)
(O)
(P)
(Q)
(R)

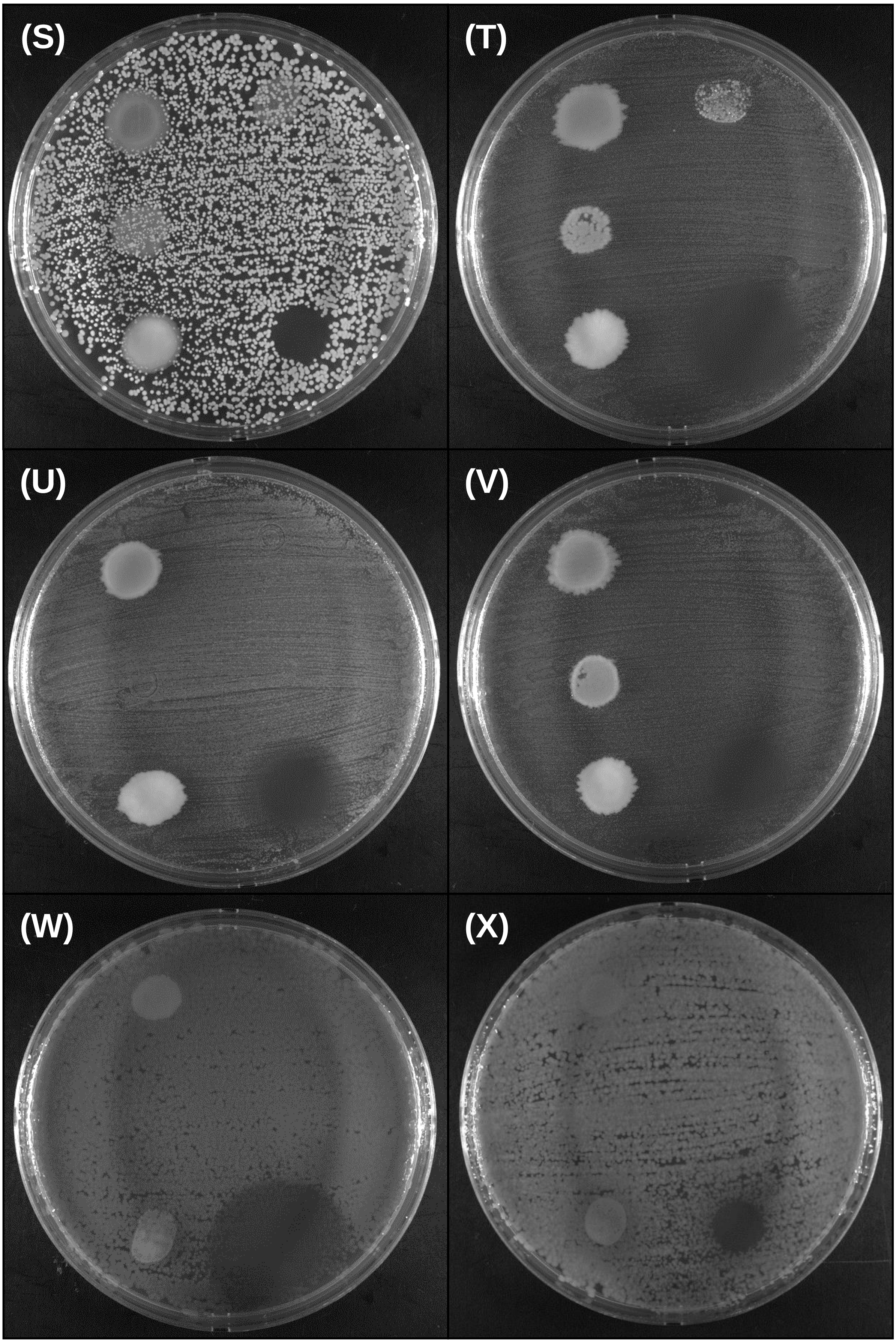
(S)
(T)
(U)
(V)
(W)
(X)

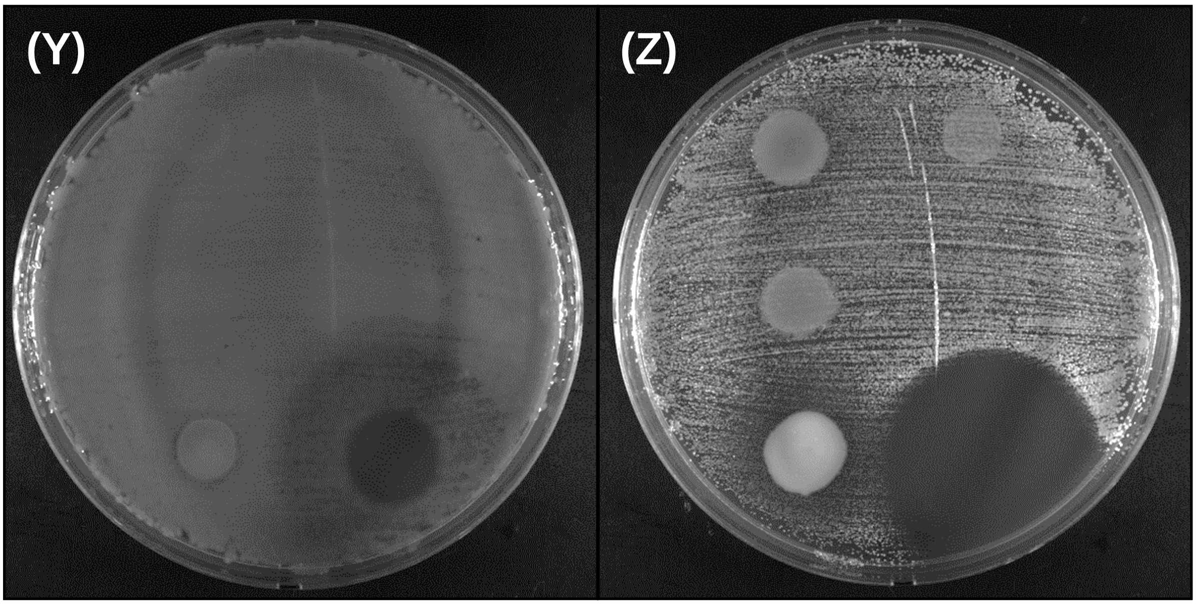

Figure S1. The drop assay of 25 types of pathogen by *Pseudomonas* sp. NIBR-H-19. (A) Schematic of the drop assay. Image was taken after 72 hour incubation at 27℃. (B) *Acinetobacter boumannii* 04 p4^{+}, (C) *A. baumannii* ATCC 17978, (D) *A. boumannii* ATCC 19606, (E) *Aeromonas hydrophilla*, (F) *Bacillus cereus* ATCC 11778, (G) *Edwardsiella piscicida* CK41, (H) *Enterococcus faecalis* ATCC 10541, (I) *Klebsiella pneumoniae* ATCC 10031, (J) *Listeria monocytogenes* ATCC 15313, (K) *Listonella anguillarum* KCTC 2711, (L) *Rhodococcus equi* ATCC 6939, (M) *Salmonella typhi* ATCC 19430, (N) *S. typhimurium* χ3339, (O) *Shigella flexneri* ATCC 9403, (P) *S. sonnei* ATCC 29930, (Q) MRSA, (R) *Staphylococcus aureus* ATCC 25923, (S) *S. haemolyticus*, (T) *Streptococcus iniae* KCTC 3657, (U) *S. iniae* GNU, (V) *S. iniae* NIFS, (W) *Vibrio cholerae* ATCC 25872, (X) *V. parahaemolyticus* ATCC 17802, (Y) *V. vulnificus* ATCC 33149, and (Z) *Staphylococcus hominis*.

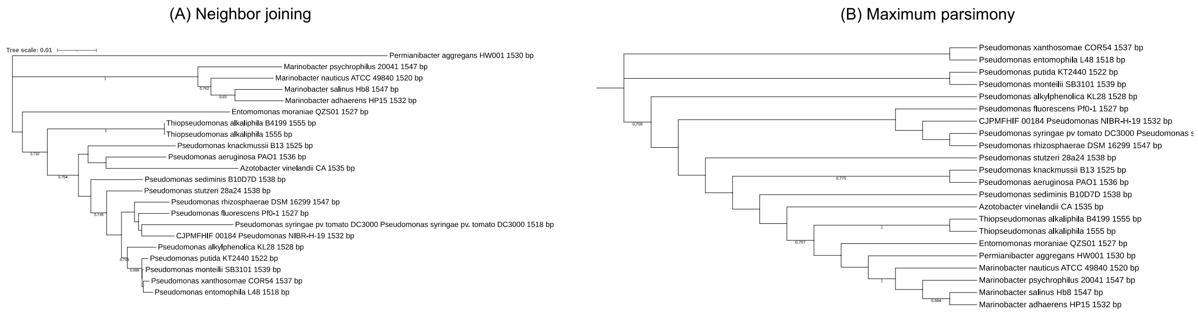

Figure S2. Phylogenetic trees with 16s ribosomal RNA sequences. (A) Tree constructed by the neighbor-joining (B) Tree constructed by the Maximum-parsimony. Bootstrap values are written in the percentage of 1000 replicates (only values > 70%).
